# Dissociations between developmental dyslexias and attention deficits

**DOI:** 10.3389/fpsyg.2014.01501

**Published:** 2015-01-12

**Authors:** Limor Lukov, Naama Friedmann, Lilach Shalev, Lilach Khentov-Kraus, Nir Shalev, Rakefet Lorber, Revital Guggenheim

**Affiliations:** School of Education and Sagol School of Neuroscience, Tel Aviv UniversityTel Aviv, Israel^1^

**Keywords:** developmental dyslexia, attention, letter position dyslexia, attentional dyslexia, dissociation, neglect dyslexia, surface dyslexia

## Abstract

We examine whether attention deficits underlie developmental dyslexia, or certain types of dyslexia, by presenting double dissociations between the two. We took into account the existence of distinct types of dyslexia and of attention deficits, and focused on dyslexias that may be thought to have an attentional basis: letter position dyslexia (LPD), in which letters migrate within words, attentional dyslexia (AD), in which letters migrate between words, neglect dyslexia, in which letters on one side of the word are omitted or substituted, and surface dyslexia, in which words are read via the sublexical route. We tested 110 children and adults with developmental dyslexia and/or attention deficits, using extensive batteries of reading and attention. For each participant, the existence of dyslexia and the dyslexia type were tested using reading tests that included stimuli sensitive to the various dyslexia types. Attention deficit and its type was established through attention tasks assessing sustained, selective, orienting, and executive attention functioning. Using this procedure, we identified 55 participants who showed a double dissociation between reading and attention: 28 had dyslexia with normal attention and 27 had attention deficits with normal reading. Importantly, each dyslexia with suspected attentional basis dissociated from attention: we found 21 individuals with LPD, 13 AD, 2 neglect dyslexia, and 12 surface dyslexia without attention deficits. Other dyslexia types (vowel dyslexia, phonological dyslexia, visual dyslexia) also dissociated from attention deficits. Examination of 55 additional individuals with both a specific dyslexia and a certain attention deficit found no attention function that was consistently linked with any dyslexia type. Specifically, LPD and AD dissociated from selective attention, neglect dyslexia dissociated from orienting, and surface dyslexia dissociated from sustained and executive attention. These results indicate that visuospatial attention deficits do not underlie these dyslexias.

## Introduction

One of the paths that the research of developmental dyslexia takes is the quest for a cognitive underlying source for developmental dyslexia. In this research we examine whether attention deficits are a source for developmental dyslexia, by searching for dissociations between the two. In our investigation we applied a neuropsychological perspective that treats both reading and attention as multifaceted constructs and as a result differentiates between types of dyslexia and between types of attention difficulties. Namely—beyond examining whether double dissociations can be found between developmental dyslexia and attention deficits, we ask more specific questions: we take specific dyslexias, analyze their possible relations to specific attention functions, and ask whether they can be dissociated from deficits in the relevant attention functions.

Dyslexia is a reading impairment that can result from brain damage (acquired dyslexia) or be present already before reading acquisition (developmental dyslexia). More than 10 types of developmental dyslexia have been identified, each resulting from deficits in different components of the reading process, and each having different characteristics (Marshall, 1984; Castles and Coltheart, 1993; Temple, 1997; Castles et al., 1999, 2006; Jones et al., 2011; Coltheart and Kohnen, 2012; Friedmann and Haddad-Hanna, 2014). Similarly, the neuroscience literature treats attention as a multifaceted system composed of several different attention networks (Posner and Petersen, 1990; Parasuraman, 2000; Tsal et al., 2005; Petersen and Posner, 2012). Tsal et al. (2005) describe four attentional subsystems (or functions) that are independent to some degree and can be localized in different anatomical loci. Therefore, in the current research we wish to explore the nature of the relation between specific types of dyslexia and specific types of attention deficits and to learn about their shared and/or separate bases.

In what follows, we briefly describe the process of normal reading that we assume and the different types of dyslexia that stem from deficits in its different components, then discuss the different attention functions and different types of attention deficits that stem from deficits in its different components, and then discuss the relation between specific types of dyslexia and specific attention deficits.

### The process of single word reading and the various dyslexia types

**Figure 1:**
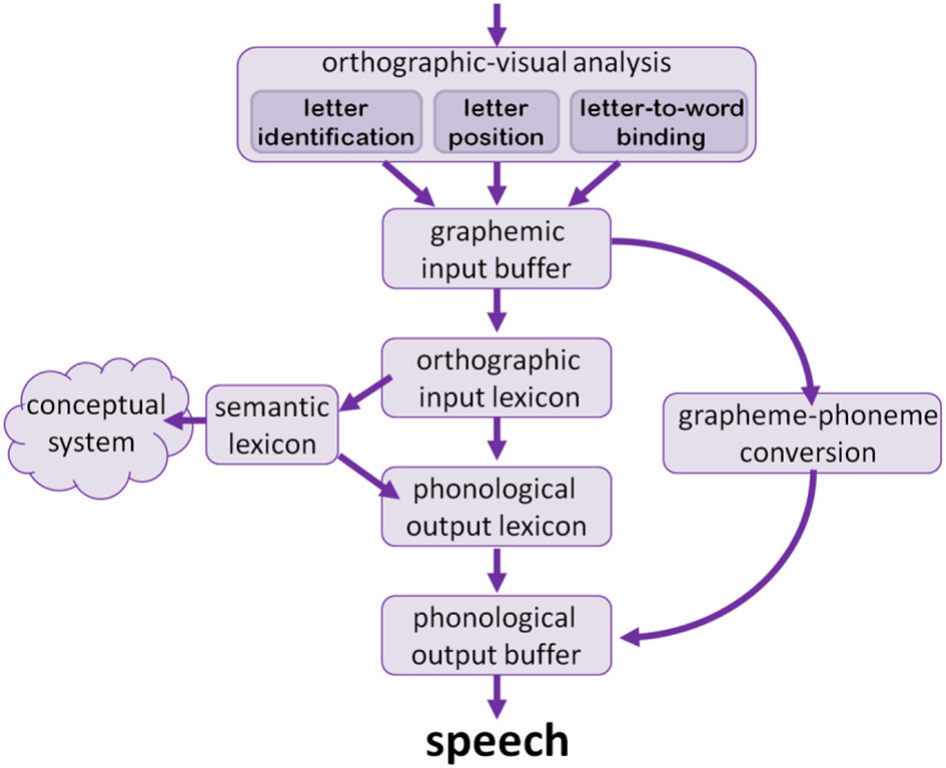
**The dual route model of single word reading**.

According to the dual-route model for single word reading (Patterson et al., 1985; Ellis and Young, 1996; Coltheart et al., 2001; Jackson and Coltheart, 2001; Castles et al., 2006; Coltheart and Kohnen, 2012, and others, see Figure 1), the early stage of reading is responsible for orthographic-visual analysis, including the identification of the abstract identity of letters in the word, the encoding of the relative position of letters within the word, and binding of letters to a word. The output of these components is held in a graphemic input buffer until it is processed in the next stages.

Each of the functions of the orthographic-visual analyzer is susceptible to a selective deficit, causing a different type of dyslexia, with different pattern of errors and effects on reading. A deficit in letter identification results in visual dyslexia, letter identification dyslexia, or letter agnosia (Nielsen, 1937; Marshall and Newcombe, 1973; Lambon Ralph and Ellis, 1997; Cuetos and Ellis, 1999; Brunsdon et al., 2006; Friedmann et al., 2012); a deficit in the encoding of letter position within words results in letter position dyslexia, characterized by letter migrations within the word (Friedmann and Gvion, 2001, 2005; Friedmann and Rahamim, 2007, 2014; Friedmann et al., 2010a; Friedmann and Haddad-Hanna, 2012, 2014; Kohnen et al., 2012).

A deficit in letter-to-word binding gives rise to attentional dyslexia, in which letters migrate between words (Shallice and Warrington, 1977; Price and Humphreys, 1993; Saffran and Coslett, 1996; Hall et al., 2001; Humphreys and Mayall, 2001; Davis and Coltheart, 2002; Friedmann et al., 2010b). A further type of dyslexia that results from a deficit at the visual analysis stage, neglect dyslexia at the word level, is characterized by neglect of one side of the word, resulting in omissions, substitutions, or additions of letters on one of the sides of the word, typically on the left side (Bisiach et al., 1986; Ellis et al., 1987, 1993; Caramazza and Hillis, 1990; Cubelli et al., 1991; Haywood and Coltheart, 2001; Arduino et al., 2002, 2003; Vallar et al., 2010).

From the orthographic-visual analysis stage, the information flows in two routes: a lexical route and a sublexical route. The lexical route starts with an orthographic input lexicon, which stores the orthographic form of words the reader is acquainted with. The information that arrives from the orthographic-visual analyzer activates an entry of a word in the orthographic input lexicon. This entry, in turn, activates an entry in the phonological output lexicon, where information about the phonology of the word is stored, including consonants, vowels, stress position, and number of syllables. This phonological information then activates the phonological output buffer, which constructs the phonological representation from the consonants, vowels, and their order, and holds the phonological information until the word is spoken. The lexical route is the fast and accurate route for reading aloud. Another branch of the lexical route arrives from the orthographic input lexicon to the semantic-conceptual system, where the information about the meaning of the written word is stored.

The other route, the sublexical route, allows the reading of unfamiliar words, by converting graphemes into phonemes. This route may cause regularization in reading irregular words (such as reading *love* to rhyme with cove and *listen* with a pronounced “t”). The correct reading aloud of such irregular words requires reading through the lexical route. Recent studies of dyslexia teach us that the sublexical route converts consonants and vowels separately (Khentov-Kraus and Friedmann, 2011), and converts graphemes with sensitivity to phonological features (Gvion and Friedmann, 2010).

Again, different types of dyslexia result from deficits in various loci in these two routes. A deficit in the lexical route results in surface dyslexia (Marshall and Newcombe, 1973; Newcombe and Marshall, 1981, 1984, 1985; Coltheart et al., 1983; Coltheart and Funnell, 1987; Howard and Franklin, 1987; Castles and Coltheart, 1993, 1996; Weekes and Coltheart, 1996; Ellis et al., 2000; Judica et al., 2002; Castles et al., 2006; Friedmann and Lukov, 2008). Because readers with surface dyslexia cannot use the lexical route to read aloud, they read via grapheme-to-phoneme conversion. As a result, their reading is slower (Zoccolotti et al., 1999), and, in the case of irregular words and words for which grapheme-to-phoneme conversion is ambiguous, also inaccurate.

An impairment in the sublexical route gives rise to phonological dyslexia, in which readers can read only via the lexical route, so they are only able to read correctly words that are already in their orthographic input lexicon, whereas they experience great difficulty in reading aloud nonwords and new words (Temple and Marshall, 1983; Glosser and Friedman, 1990; Coltheart, 1996; Friedman, 1996; Southwood and Chatterjee, 1999, 2001). This dyslexia can result from a deficit in grapheme-to-phoneme conversion or from a deficit in the phonological output buffer (Guggenheim and Friedmann, 2014). Given the special nature of the sublexical route described above, specific types of dyslexia can result from a selective deficit in reading vowel letters (Khentov-Kraus and Friedmann, 2011; Friedmann and Haddad-Hanna, 2014), or a selective deficit in some phonological features like voicing (Gvion and Friedmann, 2010).

A deficit to both the lexical and the sub-lexical reading routes results in *deep dyslexia*, which causes semantic errors in reading (reading *smile* as “laugh,” and *swam* as “swimming”), and inability to read nonwords and function words.

Thus, various types of dyslexia exist, with different patterns of errors in reading, which result from damage to different components of the reading process. Most of the subtypes of dyslexia were initially identified only in acquired dyslexia. In recent years we see more and more studies that provide robust evidence for the existence of subtypes of developmental dyslexia, which show striking similarity to subtypes of acquired dyslexia. This has been reported for developmental *surface dyslexia* (Broom and Doctor, 1995a; Temple, 1997; Masterson, 2000; Castles et al., 2006; Friedmann and Lukov, 2008), developmental *phonological dyslexia* (Broom and Doctor, 1995b; Temple, 1997; Guggenheim and Friedmann, 2014), developmental *deep dyslexia* (Stuart and Howard, 1995; Friedmann and Haddad-Hanna, 2014), developmental *letter position dyslexia* (Friedmann and Rahamim, 2007, 2014; Kohnen et al., 2012; Friedmann and Haddad-Hanna, 2014), developmental *visual dyslexia* (McCloskey and Rapp, 2000), developmental *attentional dyslexia* (Rayner et al., 1989; Shvimer et al., 2009; Friedmann et al., 2010b), developmental vowel dyslexia (Khentov-Kraus and Friedmann, 2011), and developmental *neglect dyslexia* (Friedmann and Gvion, 2002; Friedmann and Nachman-Katz, 2004).

### Attention functioning

Within the neuropsychological perspective, attention is also treated as a multifaceted construct. In the present study we adopted the model of four functions of attention proposed by Tsal et al. (2005). This model is derived from Posner and Petersen's (1990) influential theory of attention networks. The four-functions-of-attention model refers to four distinct functions within the attention regime: (a) sustained attention - the ability to allocate attentional resources to a non-attractive task over time while maintaining a constant level of performance; (b) selective (spatial) attention—the ability to focus attention on a relevant target while ignoring adjacent distracters; (c) orienting of attention—the ability to direct attention over the visual or auditory field according to sensory input, and to disengage and reorient efficiently; (d) executive attention - the ability to resolve conflicts of information and/or responses.

In a study that compared the attention functioning of children with and without ADHD (Attention Deficit Hyperactivity Disorder), Tsal et al. (2005) reported that sustained attention deficits were the most frequent deficit, which characterized many of the participants in the ADHD sample, whereas each of the deficits in selective, orienting and executive attention characterized approximately half of participants in the ADHD sample. Importantly, Tsal et al.'s study showed that ADHD can entail deficits in any single (or combination of) attention function/s. Thus, different children with ADHD can have divergent clusters of attention deficits.

### On the relation between dyslexia and attention deficits

Attention disorders and reading disorders are often reported to co-occur (e.g., August and Garfinkel, 1990; Semrud-Clikeman et al., 1992; Snider et al., 2000; Willcutt and Pennington, 2000). Some researchers suggest that this co-occurrence is principled, and that attention lies at the basis of reading, and hence, attention deficits may underlie dyslexia. For example, Clark (1999) suggested that attention should be engaged at the word-target location before a saccade can be made to that location. Hoffman and Subramaniam (1995) have shown that spatial attention is a crucial mechanism in generating voluntary saccadic movements. Thus, visuospatial attention initiates the saccade, and the programming of the next saccade begins when visual attention shifts from the fovea toward the next word into the parafoveal area (Clark, 1999).

Other researches provide less-specific approaches to the relation between attention and reading but claim that attention is crucial for reading. For example, Reynolds and Besner (2006) suggest that attention is critical for translating print into speech, and Shaywitz and Shaywitz (2008) claim that attention has a role in reading, and that deficient attention may cause reading difficulties. Several previous studies reported certain attention deficiencies in children with dyslexia compared with typically developed children (Slaghuis et al., 1993; Casco and Prunetti, 1996; Casco et al., 1998; Vidyasagar and Pammer, 1999). For instance, Facoetti and his colleagues found that children with dyslexia (without specifying the types of dyslexia) did not benefit from exogenous (peripheral) precues although they did demonstrate improved performance when endogenous (central) precues were introduced (Facoetti et al., 2000, 2003a,b). According to these studies, difficulties in spatial attention (orienting and/or selective) may serve a causal role in dyslexia.

However, comorbid occurrence of two deficits is still not necessarily indicative of a principled relation between them. In the current study we aim to examine whether co-occurrences of reading and attention difficulties indicates a causal relation between the two. The way neuropsychology usually approaches questions of relations between modules and functions is by searching for dissociations and double dissociations: if a double dissociation between dyslexia and attention deficits is found, then reading and attention are independent modules that can be selectively impaired, and an impairment in one does not result from an impairment in the other. Thus, this study searched, first, for double dissociations between developmental dyslexia and attention deficit in general. The next stage is aimed to explore the finer relations between specific types of dyslexia and attention, asking whether in cases of comorbid impairments, specific dyslexias are linked to certain specific attention deficits. To the best of our knowledge, no study has tested the relations between subtypes of developmental dyslexia and subtypes of attention difficulties.^2^

### Dyslexias suspected to have attentional basis

Three types of peripheral dyslexia that affect many Hebrew readers with developmental dyslexia present characteristics that seem to be related to attention, and are hence the best candidates for having attention deficits of some sort at their basis. One is *letter position dyslexia* (LPD, Friedmann and Gvion, 2001, 2005; Friedmann and Rahamim, 2007, 2014; Friedmann et al., 2010a; Friedmann and Haddad-Hanna, 2012, 2014; Kohnen et al., 2012; Kezilas et al., 2014). LPD is characterized by transpositions of middle letters within words. According to some analyses, it results from a difficulty in attention allocation to letters, whereby attention is allocated to the first and final letters in the word, and then to all middle letters together. This creates illusory conjunctions between middle letters and their positions (Friedmann and Gvion, 2001). The question that immediately arises is whether this is a general visuo-spatial attention of the type that is measured in attention tasks, or whether this is an orthographic-specific attention function that is specifically harnessed to reading.

*Attentional dyslexia* is another dyslexia that might stem from a deficit in attention (as the title that Shallice and Warrington selected for the 1977 article in which they first reported this type of dyslexia already suggests: “The possible role of selective attention in acquired dyslexia”). Attentional dyslexia is an impairment in binding letters to words, which results in migrations of letters between words (Shallice and Warrington, 1977; Warrington et al., 1993; Saffran and Coslett, 1996; Hall et al., 2001; Humphreys and Mayall, 2001; Davis and Coltheart, 2002; Mayall and Humphreys, 2002; Friedmann et al., 2010b). A possible attentional approach for attentional dyslexia would ascribe it to a deficit in selective attention that hampers the ability to glue letters to words, or the ability to focus on the target word and attenuate neighboring words.

Another theoretically possible point of contact between dyslexia and attention is *neglect dyslexia*, in which the deficit is related to a specific difficulty in shifting attention to one of the sides of the word, usually its left side. The main types of errors in this dyslexia are omissions, substitutions, and additions of letters in the neglected side (Vallar et al., 2010; and see Friedmann and Nachman-Katz, 2004; Nachman-Katz and Friedmann, 2007, 2008, 2009, 2010; Friedmann and Haddad-Hanna, 2014, for the developmental form of this dyslexia). A natural place to look for an attentional source of this dyslexia would be in orienting of attention. And again, the question is whether this is a general visuo-spatial attention or an orthographic-specific one.

Finally, a different sort of relation between attention and dyslexia may characterize *surface dyslexia*. As explained above, surface dyslexia is a deficit in reading via the lexical route that results in reading via the sublexical route. One may imagine several mechanisms in which attention deficits may give rise to surface dyslexia errors. One is a general one - given difficulties in sustained attention during childhood and during the time of learning to read, children may not be able to attend to classes and devote resources to learning to read, reading, and doing homework. As a result, they might not be familiar with many written words, their lexicon would be impoverished, and their reading would have to rely on the sublexical route^3^. Similar indirect reduction of time allotted to reading may be caused by deficits in other attention functions such as selective attention, which affect the ability or motivation of a child to cope with the situation of reading in general. A more specific effect was suggested by Valdois and collegues (Valdois et al., 2004; Bosse et al., 2007). According to Valdois et al., an impairment of visual attention that reduces the visual attention span – the number of elements that can be identified in parallel—could also lead to reading letter-by-letter in a way typical to surface dyslexia^4^. Finally, it is also possible to imagine a more specific mechanism related to executive attention, assuming that executive attention is responsible for keeping the reader on the lexical route and resolving conflicting inputs that come from the output of the parallel sublexical route.

In the second part of this research we therefore assessed these specific questions on the fine relations between different types of dyslexia and specific attentional functions.

### Some evidence to the dissociability of developmental dyslexia and attention deficits

One source of evidence to the dissociability of dyslexia and ADHD comes from the differential effect that methylphenidate (MPH) has on the two. MPH is the most commonly used drug treatment for ADHD. Keidar and Friedmann (2011) assessed whether individuals with developmental dyslexia and ADHD whose attention deficits are relieved by MPH also show reduced rates of errors in reading with MPH. They tested 20 Hebrew-speaking participants with attentional-based dyslexia (mainly LPD and attentional dyslexia) and ADHD, once with and once without MPH. The results were that even though MPH positively affected their performance in at least one of the attentional functions (sustained, selective, orienting, or executive attention), it did *not* improve their reading accuracy. All of these participants had LPD, and many of them also had attentional dyslexia, but still their rate of migrations between words and within words was not affected by MPH. This study already provides some evidence that reading and attention systems are separate, and that the deficit that underlies LPD and attentional dyslexia is orthographic-specific rather than resulting from a general attentional deficit. Had the attention deficit been the source of the reading impairment, we would have expected improved attention to also reduce reading errors.

Another type of evidence suggesting that the deficit in LPD and in neglect dyslexia is orthographic-specific rather than resulting from a general attentional deficit comes from the differential effect of dyslexia on the reading of words and numbers. Friedmann et al. (2010a) reported on ten individuals with LPD who made many migration errors of letters within words, but their performance on multi-digit number reading was good: they read numbers without migration errors, and not differently from the control participants. Had attention been the source of the deficit in reading, we would expect all kinds of stimuli to be affected by it, including numbers, and not only words. Similarly, Friedmann and Nachman-Katz (2004) and Nachman-Katz and Friedmann (2008) reported on 21 individuals with developmental neglect dyslexia who made neglect errors on the left side of words, but not on the left side of multi-digit numbers. Such a dissociation between word and number reading is inconsistent with a general visuo-spatial attention deficit, which should have affected both types of stimuli.

Finally, Collis et al. (2013) recently examined the performance in a partial report task of adults with developmental dyslexia who make letter position errors and migrations between words (parallel to LPD and attentional dyslexia). They compared the participants' performance on strings of letters and symbols (as well as digits), and found that the participants with developmental dyslexia performed poorer than the control participants, but their deficit was limited to letter strings, and did not affect symbol strings. These findings suggest that the dyslexic participants did not suffer from a general visuo-spatial deficit in the visual attentional window, but rather from a deficit that was limited to orthographic material.

In this study we examined the relation between developmental dyslexia and attention deficits from another perspective, by systematically examining developmental dyslexia types and specific attention difficulties. We aimed to identify, at the cognitive level, the bases of different types of reading difficulties and different types of attention deficits. We assessed the reading and attention of all the participants. Firstly, we asked whether reading and attention are separate cognitive modules. Then, we asked whether participants with specific types of dyslexia share a specific attention deficit. The rationale was that if we can identify cases of dissociation between dyslexia and attention deficits, and specifically, if we can identify individuals with dyslexia that has a suspected attentional cause who do not have a visuo-spatial attention deficit, attention cannot be the underlying cause for this dyslexia.

## Methods

### Participants

The participants we report below are 110 Hebrew-readers with either dyslexia or attention deficit, 65 of them are children and adolescents (age ranges 10;0–17;0, *M* = 13;2, 35 girls and 30 boys) and 45 are adults (with ages ranging 18;1–42;0, *M* = 28;2, 23 women and 22 men). We only included children older than 10 years of age, to make sure that they have already had enough time to fully acquire reading and to establish an orthographic lexicon. For all of the participants, the reading and attention deficits were developmental: none of them had history of brain lesion, neurological disease, or loss of consciousness. For all of them Hebrew was the first language, and the first language in which they learned to read.

Most of the participants responded to ads that invited volunteers with both reading and attention deficits and a few of them responded to ads that looked for individuals with difficulties in at least one of the above domains. The Tel Aviv University and the Ministry of Education Ethics Committees approved the experimental protocol.

All participants completed two extensive test batteries: a reading battery and an attention functioning battery. Because we were only interested in dissociations between reading and attention deficits, we only included in the study participants who had a deficit in at least one of the domains: dyslexia, or attention deficits, or both.

### Reading assessment

To evaluate the oral reading of each participant and to determine which type of dyslexia each participant had, we tested each of the participants using the TILTAN screening test (Friedmann and Gvion, 2003), which was developed to identify subtypes of dyslexia in Hebrew. The screening test includes oral reading of 136 single Hebrew words (2–11 letters long), 30 word pairs (3–6 letters long), and 40 nonwords (3–6 letters long). According to the error types in the screening test, we ran additional tests to each participant for the types of dyslexia that emerged from the reading aloud test. These tests are described below for each type of dyslexia.

The word list in the screening test included words of various types that can reveal the different types of dyslexia: 65 migratable words—words in which middle letter migration creates another existing word, for the identification of letter position dyslexia; 104 words for which omission, substitution, migration, or addition of a vowel letter creates another existing word, for the identification of vowel letter dyslexia; 136 words for which neglect of the left side of the word yields another existing word, for the identification of neglect dyslexia, and 108 words for which right neglect errors create an existing word; 84 irregular words and potentiophones for the identification of surface dyslexia; 57 morphologically complex words for deep dyslexia and phonological dyslexia; and 26 abstract nouns and 28 function words, for deep dyslexia. All the words were sensitive to visual dyslexia, as each words had more than six orthographic neighbors.

The 40 nonwords were included for the identification of impairments in the sublexical route, in phonological dyslexias or vowel dyslexia, and deep dyslexia, but also contained migratable nonwords and words that created existing words by substitution, omission, or addition of letters, and where hence also sensitive to various impairments at the orthographic-visual analyzer (letter position dyslexia, visual dyslexia, neglect dyslexia). The list of 30 word pairs was created so that between-word migrations created other existing words, for the identification of attentional dyslexia.

On the basis of this test, we determined whether a participant had normal reading or whether s/he had dyslexia, and if s/he had dyslexia, which types of dyslexia were suspected based on the error pattern s/he showed and the factors that affected her/his reading (frequency effect, word length effect, lexicality effect, etc.). Impaired performance in the screening task, as well as on each of the further reading tasks, was determined according to the comparison of the participant's reading to an age-matched control group. The control groups were collected in previous studies and throughout the development of the test batteries. The control group for the adult participants included 372 adults, the children control groups included at least 20 children in each age group. Skilled readers after 8th grade showed identical reading pattern to the adult control group. Each participant's performance was compared to the control group using the Crawford and Howell's (1998) *t*-test for the comparison of the performance of a participant with a control group. An impaired performance was defined as performance that was significantly below the control, with *p* < 0.05. The type of dyslexia was determined using the same procedure and statistical test, applied to the various types of errors. We determined that a participant had a certain dyslexia if s/he made significantly more errors of the relevant type compared to the control group, and performed significantly poorer than the control group in the relevant reading tests. We only included in the no-dyslexia group individuals who performed within the normal range in all the reading tests. Unclear cases, with performance that was marginally different from that of the control group, and hence could not form a clear case of dissociation, were excluded from the study.

Letter position dyslexia was determined according to the number of letter position errors in reading migratable words (See Appendix C for Hebrew examples of the words of the various types and types of errors).

Attentional dyslexia was determined according to the number of between-word errors, including between-word migrations and between-word letter omissions, in reading migratable word pairs.

Left neglect dyslexia was determined according to letter errors (substitutions, omissions, and additions) that occurred predominantly on the left side of the words (see Friedmann and Nachman-Katz, 2004; Friedmann and Gvion, 2005; Nachman-Katz and Friedmann, 2007, 2008, 2009, 2010; Reznick and Friedmann, 2009, for a description of the manifestation of neglect dyslexia in Hebrew readers).

Surface dyslexia was determined according to the number of reading errors that resulted from reading via the sublexical route rather than via the lexical route, which caused regularization errors in irregular words and potentiophones.

Vowel dyslexia was determined according to the number of vowel letter errors (migrations, substitutions, omissions, and additions) in words and nonwords.

We then further tested the participants' reading in additional tests from the TILTAN battery that were specific to different types of dyslexia that emerged from the screening test, in order to establish decisively the type of dyslexia each participant had. In these additional tests, reading aloud was done without time limit, and the participants were requested to read aloud as accurately and as quickly as possible. The first responses were counted, even when they were later self-corrected. In the lexical decision and the comprehension tasks, the participants were requested to perform the tasks in silent reading, without sounding out the words they read.

The results of each participant in each of the further reading tasks was compared to those of age-matched controls. In the reading aloud tasks, the number of errors of each type (reading via the sublexical route, vowel omission, substitution, addition, migration, consonant omission, substitution, addition, migration, letter neglect on the left, migrations between words, voicing errors, semantic errors) was compared to the number of these errors in the control group. In the lexical decision and comprehension tasks, the percentage of correct responses was compared to that of the control group.

#### Letter position dyslexia

To establish the diagnosis of letter position dyslexia, which is characterized by letter migrations within words, we used tasks that tested the participants' oral and silent reading of words that are most sensitive to this dyslexia—migratable words. These are words in which migration of middle letters within the words creates another existing word (such as cloud-could, parties-pirates, casual-causal).

The *reading aloud* task for LPD included 232 migratable words of 4–7 letters (*M* = 4.9, *SD* = 0.9). In 87 of these words, a middle migration that involves a vowel letter and a consonant letter creates another existing word, and in 163 words a middle migration that involves two consonant letters creates another word. (For an English example, the word *stops* has a potential for transposition of two consonant letters- *t* and *p*, creating the words *spots*, and the word *form* has a potential for migration that involves a vowel—a transposition of *o* and *r* would create the word *from*).

Additional tasks involved *same-different decision* in which the participant was presented with 60 word pairs, half of which differed in middle letter order (clam-calm), and was requested to determine whether the words in the pair are same or different; *lexical decision task*, in which the participants saw 60 items, half of them words and half migratable nonwords (pecnil) and were requested to determine whether the item was a word; and a *reading comprehension task* that included 50 triads. Each triad consisted of a target migratable word, and two words to choose from: one word that is semantically associated with the target word, and one that is semantically associated with a word that can result from a transposition of middle letter (dairy → milk, notebook). The participants were requested to circle the word that is semantically associated with the target word.

#### Attentional dyslexia

To establish the diagnosis of attentional dyslexia, characterized by migrations of letters between neighboring words (and by omissions of an instance of a letter that appears in two neighboring words in the same position), the participants read aloud additional lists of word pairs and a list of nonword pairs.

The *word pair* list included 120 word pairs of 2–7 letters (*M* = 4.8, *SD* = 1). All these word pairs were migratable, namely, for each of them, migration of a letter from one word to the other, preserving the within-word position, creates another existing word (e.g., *mild wind* in which between-word migration can create *wild mind*). The *migratable nonword pair* list included 30 3-letter nonword pairs in which letter migration between words would result in existing words.

#### Neglect dyslexia

Identification of neglect dyslexia was based on an analysis of the position of consonant letter errors (substitutions, additions, and omissions) in the three subtests of the reading aloud screening task, as well as three additional tasks: reading aloud of words and nonwords that share the right side with other words, and lexical decision.

The *oral reading of words* for neglect dyslexia included 100 words in which substitution, omission, or addition of a letter on the left side created another existing word (*rice*→ nice, price, ice). The list for *oral reading of nonwords* for neglect dyslexia included 30 nonwords that differ from existing words in the left letter (*netter*). The *lexical decision* task included 50 nonwords that differ from existing words in the left letter (*diraffe*), as well as 40 existing words.

#### Surface dyslexia

***Surface dyslexia test: Reading aloud of potentiophones***. To establish surface dyslexia, which is characterized by reading via the sublexical route, the participants read aloud 78 potentiophonic words, 2–6 letters long (*M* = 3.7 letters, *SD* = 0.8). Potentiophones are words whose reading via grapheme-to-phoneme conversion creates another existing word (like *now*, which can be read via grapheme-to-phoneme conversion to sound like “know,” Friedmann and Lukov, 2008). Such words are the most sensitive stimuli to detect surface dyslexia because, like irregular words, their correct reading requires the lexical route. They are more sensitive to surface dyslexia than other irregular words because reading them via the sublexical route results in another word, and hence the reader cannot know that the word was read erroneously.

***Pseudo-homophone lexical decision***. The lexical decision task for surface dyslexia contained 66 word pairs. Each pair included a word spelled correctly and its pseudo-homophone (e.g., knife-nife). For each pair, the participants were requested to circle the word that was spelled correctly.

***Homophone-potentiophone written word comprehension***. The reading comprehension task included 40 triads. Each triad consisted of a target word, and two words to choose from: one word that is semantically associated with the target word, and a homophone or a potentiophone of the associated word (e.g., bottle—bear beer). The participants were requested to circle the word that is semantically associated with the target word.

#### Vowel dyslexia

To establish the diagnosis of vowel dyslexia, characterized by substitutions, omissions, additions and migrations of vowel letters, the participants performed two additional tasks of lexical decision and word comprehension.

***Lexical decision***. The vowel dyslexia lexical decision task contained 80 items: 45 nonwords in which a vowel error creates existing Hebrew words and 35 existing words—16 of which included a vowel letter and 19 without vowel letters. The items in the task were 2–8 letters (*M* = 4.8, *SD* = 1.13). The participants were requested to silently read each word and to circle the words that exist in Hebrew.

***Written word comprehension***. The reading comprehension task for vowel dyslexia included 52 triads. Each triad consisted of a target word (3–6 letters long, *M* = 4.4, *SD* = 0.75), and two to four words to choose from: one word that is semantically associated with the target word, and the rest are words that are semantically associated with words that can result from a vowel error in the target word (form → shape, to, ranch). The participants were requested to circle the word that is semantically associated with the target word.

### Attention assessment

Attention functioning was assessed by using four computerized neuropsychological tasks, serving as indicators of performance in each of the attention functions (Tsal et al., 2005). The four attention tasks enable us to assess the attentional profile of each participant. The performance of each participant in each of the above tests was compared to that of an age-matched control group. The control group for the adult participants included 300 adults, and the children control groups included at least 30 children in each age group collected throughout the development of the test battery. A deficit in sustained, selective, or executive attention was defined in cases where an individual's performance was located in the lowest five percentages of the distribution of her/his age-matched control group, that is, when ≤−1.645. In orienting attention there are two different possible deficits: a deficit in disengagement of attention (when invalid cue caused a large decrease in performance) and a deficit in automatic orienting of attention (when a valid cue was not effective and did not improve performance). The former was defined in cases where the performance was located in the lowest 5% of the distribution, that is, when ≤−1.645 and the latter was defined when the performance was located in the highest 5% of the distribution, that is, when ≥ 1.645. Each attention test starts with a short practice block and the test lasts approximately 12 min. The task that assessed sustained attention was always administered as the first task. The other three attention tasks were administered in a counter-balanced order.

#### Sustained attention

For sustained attention, we used a Conjunctive Continuous Performance Test (CCPT). Participants were presented with a long series of stimuli but were instructed to respond to a single reoccurring pre-specified target (a red square) while withholding responses to all other, non-target stimuli. There were four possible shapes (square, circle, triangle, and star) and four possible colors (red, blue, green, and yellow). As soon as a target appeared the participant was requested to press the spacebar. Using a low rate of target stimuli (30%) and varying the inter-stimulus interval (ISI), this task maintains a high demand on sustained attention but minimizes the involvement of other cognitive factors. Standard deviation of mean RT of target trials served as the measure of sustained attention (Shalev et al., 2011; Stern and Shalev, 2013).

#### Selective (spatial) attention

For selective attention, we used a conjunctive search task (Tsal et al., 2005). Participants were instructed to search for a target stimuli appearing among distracters. The displays varied in their set size (i.e., the number of distractors), enabling estimation of the effect of attentional load on performance. The participant was instructed to fixate on a central fixation point which was followed by a display of items. The participant was requested to decide whether the display contained the target—a blue square among the distractors (blue circles and red squares). The target appeared in 50% of the displays. If a target was detected the participant had to press the “L” key and if the target was absent then s/he had to press the “A” key. The slope of the search graph reflected the efficiency of spatial selective attention.

#### Orienting attention

For orienting of attention, we used a peripheral cueing paradigm (Posner et al., 1980) with an exogenous cue (Jonides, 1981). Participants had to discriminate a stimulus—a triangle or a circle—preceded by an abrupt onset at either the target's location (valid cue) or the opposite side of fixation (invalid cue). When the target was a triangle the participant had to press the “L” key and when the target was a circle s/he had to press the “A” key. The difference in performance between valid and invalid trials indicates the ability to orient attention and efficiently disengage from irrelevant locations (Tsal et al., 2005).

#### Executive attention

For executive attention, we used a Location-Direction Stroop-like task (Stroop, 1935) with a spatial aspect. Participants had to respond either to the location or the direction of an arrow (in different blocks) appearing on the screen, while ignoring the other irrelevant dimension. Half of the stimuli were congruent trials (that is, the location on the screen and the direction of the arrow match; i.e., an arrow presented below fixation pointing downwards) and half of them were incongruent (i.e., an arrow presented above fixation pointing downwards). In the first two blocks of the tasks participants were requested to judge the location of the arrow (relative to the fixation point; if it is presented above the fixation they had to press “L” and if it is presented below the fixation they had to press “A”) and in the last two blocks they were requested to judge its direction (Tsal et al., 2005). The widely-used interference effect in such tasks reflects the extent to which conflicting irrelevant information is being effectively suppressed.

## Results

### Part A: dissociations between dyslexia and attention deficits

One of the most fundamental tools in the neuropsychological toolbox is that of double dissociation. Such a condition in which one person has impairment in cognitive ability A but has normal performance on B, and another person with the opposite dissociation, impairment in cognitive ability B but with normal performance on A, suggests that A and B are separate modules. Thus, if we are able to identify a double dissociation between dyslexia and attention deficits, we can demonstrate that neither of them underlies the other. Specifically for this special issue, we will be able to answer the question as to whether attention deficits underlie developmental dyslexia in “NO.”

As shown in Tables 1, 2, we identified 55 participants who showed a double dissociation between reading and attention functions: As summarized in Table 1, 28 had dyslexia with normal attention functioning (12 children and 16 adults), and 27 had deficits in at least one attention function, with normal reading (10 children and 17 adults). Importantly, various types of dyslexia showed dissociations with attention: among the participants with dyslexia who had spared attention abilities there were 21 individuals with letter position dyslexia, 13 with attentional dyslexia, and 2 with neglect dyslexia, all dyslexias that have been linked by some to attention functions, as well as 12 participants with surface dyslexia, 11 with vowel dyslexia, and one woman with phonological buffer dyslexia. Appendix A details the relevant error rates in reading aloud for each of these participants- for each participant, errors of each type that occurred at a rate significantly (p < 0.05) higher than that of an age-matched control group appear under the relevant dyslexia type. Empty cells in Appendix A indicate no errors or a small percentage of error within the normal range for the relevant error (average percentage and *SD* of each type of error for each control age group appear in the bottom of each dyslexia column).

**Table 1:** **The types of dyslexia among individuals with intact attention and impaired reading (*n* = 28)**.

**Dyslexia**	**Number of participants with intact attention who showed these dyslexias**
LPD	5
LPD, attentional dyslexia	3
LPD, attentional dyslexia, surface dyslexia	4
LPD, attentional dyslexia, vowel dyslexia, surface dyslexia	3
LPD, attentional dyslexia, vowel dyslexia	1
LPD, attentional dyslexia, neglect dyslexia, vowel dyslexia, surface dyslexia	1
LPD, surface dyslexia	2
LPD, vowel dyslexia	1
LPD, vowel dyslexia, surface dyslexia	1
Attentional dyslexia, neglect dyslexia	1
Vowel dyslexia	4
Surface dyslexia	1
Phonological buffer dyslexia	1

**Table 2:** **The various attention deficits among individuals with intact reading and impaired attention (*n* = 27)**.

**Attention deficits**	**Number of participants with intact reading who showed these attention deficits**
Sustained	5
Orienting	3
Executive	1
Selective	1
Sustained and orienting	4
Sustained and executive	4
Sustained and selective	3
Orienting and executive	2
Selective and executive	1
Sustained, orienting, and executive	1
Sustained, selective, orienting, and executive	2

These results suggest that each of these types of dyslexia can be dissociated from attention deficits, indicating that reading and attention are separate, and that attention deficits do not underlie these dyslexias.

As summarized in Table 2, it is also clear that each of the four tested attention functions could be impaired without giving rise to dyslexia. Among the 27 participants with attention deficit whose reading was intact there were 19 with deficient sustained attention, 7 with deficient selective attention, 12 with deficient orienting attention and 11 with executive attention deficit (the detailed Z-scores for each of these participants in each of the tasks are presented in Appendix B). Importantly, we demonstrated that individuals who suffer from a deficit in any of the four functions of attention (sustained, selective, orienting, and executive) may still show preserved reading. These findings further support the claim that attention deficits are not necessarily related to dyslexia.

### Part B: finer grained observations: types of dyslexia and types of attention deficits

Considering the possible connections between specific dyslexias and specific attention deficits, one can ask whether when an individual has both dyslexia and attention deficit, there are consistent relations between the type of dyslexia and the type of attention function that is impaired.

As we explained in the Introduction, several possible specific relations between dyslexia and attention deficits can be inferred from the assumption that reading and attention deficits are the result of the same core deficit. The possible connections that we examined were between letter position dyslexia and selective attention, between attentional dyslexia and selective attention, between neglect dyslexia and orienting of attention, and between surface dyslexia and sustained or executive attention. We have already seen in Section A that dyslexia can be dissociable from attention deficits altogether, and hence, we can also conclude that these types of dyslexia can be dissociated from attention deficits. In the data summarized in Table 3 we are able to explore, for individuals who have both dyslexia and attention deficit, whether the witnessed attention function that was impaired was the one suspected under a general attentional hypothesis for each dyslexia with possible attentional bases.

**Table 3:**
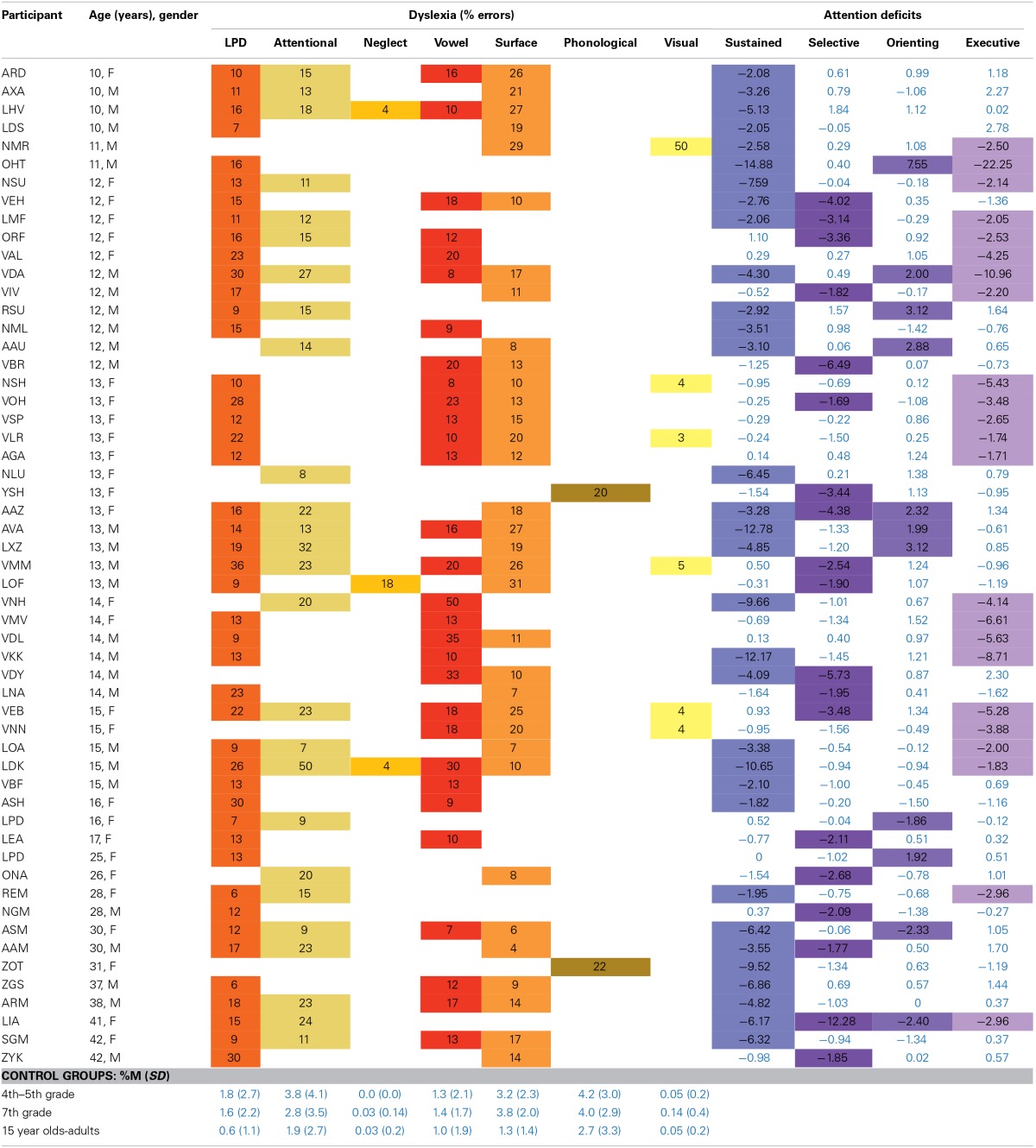
**Types of dyslexia and types of attention deficit for the participants who had both dyslexia and attention deficits**.

*Each row represents a different participant (n = 55)*.

*^a^In the attention tasks, Z scores smaller than −1.645 reflect deficient functioning. In the case of orienting attention Z scores smaller than −1.645 reflect deficient disengagement of attention and Z scores larger than +1.645 reflect deficient automatic orienting. That is, the colored cells represent performance level below the cutoff of 0.05*.

*^b^In the reading tasks, all the error rates of the various types presented in the table are significantly larger than the age-matched control groups and indicate a dyslexia of the relevant type. Empty cells indicate that there were no relevant errors at all or that the relevant error rate was within the normal range, in most cases less than 1%. The percentages of errors that appear in the table for each dyslexia are: for LPD—migrations in migratable words (except LEA, VIV, LNA, and ZYK whose error rates refer to their migrations in nonwords), for attentional dyslexia- migrations between words in migratable word pairs, for neglect—consonant omission and substitution on the left of the word, for vowel dyslexia—vowel letter omission, substitution, migration, or addition in nonwords (except NML, ASH, VKK, ORF, AVA, ARD, and LDK whose error rates refer to their vowel letter errors in words), for surface dyslexia—sublexical reading in irregular words and potentiophones, for phonological dyslexia—errors in nonwords, and for visual dyslexia—consonant omission, substitution, or addition (for NMR, who has visual-analyzer-output dyslexia, the error rates also included vowel letter errors and letter migrations)*.

*^c^As can be seen when comparing the error rates of the dyslexics to those of the controls, although, 4% neglect errors seem like a low error rate, unimpaired readers make almost no such errors and hence 4% errors (5 or 6 consonant errors on the left out of the 136 words) already signifies a significant deficit, which is supported by the additional reading tests. The same holds also for visual dyslexia, where 3% visual errors (4 consonant omission, substitution, or addition errors out of the 136 words) already indicates a significant deficit*.

Starting with letter position dyslexia, where we look for relations to a selective attention deficit, Table 3 shows that even in cases where both reading and attention are impaired, LPD does not necessarily appear with selective attention deficit. In our results, summarized in Table 3, 30 individuals had LPD but no selective attention deficit. A broader look at the other attention functions indicates that there was no single attention function that was impaired for all the individuals with LPD who also had an attentional deficit.

Similarly, attentional dyslexia can also be thought to stem from a deficit in selective attention. Table 3, however, reports on 18 individuals with attentional dyslexia who had an attention deficit but no selective attention deficit.

As for neglect dyslexia, the suspected attention function would be orienting of attention. However, the results in Table 3 include three individuals with developmental neglect dyslexia, and neither of them had a deficit in orienting of attention.

Finally, considering surface dyslexia, we suggested that a deficit in sustained attention can cause a chain of events following which children will have more limited exposure to reading, and read via grapheme-to-phoneme conversion, rather than via the lexical route. This mechanism as a basis for surface dyslexia is also not supported by our results: In Table 3 we report 15 individuals with surface dyslexia who had an attention deficit but intact sustained attention, and 13 individuals with sustained attention deficit, who did not have surface dyslexia. As for the hypothesis according to which executive attention underlies surface dyslexia, there were 10 participants with executive attention deficit without surface dyslexia, and 21 participants with surface dyslexia without executive attention deficit.

Additionally, three participants had phonological dyslexia. It may be suggested that a deficit in grapheme-to-phoneme conversion may be related to a difficulty in the serial shift of attention from one letter to the next. Such attention function may be supported by orienting of attention. This suggestion is not supported by the findings, however. There were two phonological dyslexics who also had attention disorders: one had only selective attention deficit and one had only sustained attention deficit. More importantly, in Part A we reported a woman who had phonological (buffer) dyslexia with completely normal attention functions. Similarly, no specific attention deficit was found for vowel dyslexia or visual dyslexia.

## Discussion

An important part of the quest into the nature of developmental dyslexia is the search for underlying causes for dyslexia. Often such causes are searched within the general cognitive abilities, and one such candidate is attention. In this research we explored the question of the relation between attention deficit and dyslexia from a neuropsychological perspective that takes into account the existence of various types of dyslexia and of various types of attention deficits. As a first step, we established a double dissociation between dyslexia in general and attention deficits in general in 55 individuals. We showed children, adolescents, and adults who had dyslexia (of any type) without attention deficits (all four attention functions were normally functioning). We then showed children, adolescents, and adults who had attention deficits (of any of the four types) without dyslexia (reading at the single word level was completely normal). Such double dissociation already indicates that attention cannot be the source of dyslexia.

However, we sought to be more specific in this study, and looked for the fine relations, or lack thereof, between specific types of dyslexia and specific attention functions.

Starting with *letter position dyslexia*, an imaginable attentional source would be selective attention: if an individual cannot focus attention on a restricted area, several letters could be perceived together in the attended area, and as a result their positions may be misperceived. The results, however, do not support such account. We have seen 21 individuals with LPD who had no attention deficits, and 7 individuals with a deficit in selective attention who had no dyslexia, including no LPD. Even in cases where both reading and attention are impaired, LPD does not necessarily appear with selective attention deficit. In our results, 30 individuals had LPD but no selective attention deficit. A broader look at the other attention functions indicates that there was no single attention function that was impaired for all the individuals with LPD who also had an attentional deficit.

Similarly, *attentional dyslexia* can be thought to stem from a deficit in selective attention. Here the suspected mechanism would be that a deficit in attenuation of the neighboring words would result in letters from the neighboring words being perceived with the target words. Here, again, our results do not support such an underlying basis for attentional dyslexia: firstly, in Section A we reported on 13 individuals who had attentional dyslexia without any attention deficit, and 7 individuals with selective attention deficit without attentional dyslexia. Secondly, we reported on 18 individuals with attentional dyslexia who did have an attentional deficit but no selective attention deficit.

In fact, these findings indicate that even the dyslexia that Shallice and Warrington (1980) termed “attentional dyslexia,” is actually not attentional in nature, and can occur in individuals with no general visuo-spatial attention deficit.

Additionally, one may take the dissociations found between letter position dyslexia and attentional dyslexia as an additional indication that a general deficit in selective attention cannot be the source of these dyslexias. Had selective attention deficit been the source of both these dyslexias, we would expect them to always appear together. However, in this study there are 23 individuals with letter position dyslexia who did not have attentional dyslexia, and 4 individuals with attentional dyslexia who did not have letter position dyslexia. This double dissociation was also found in previous studies of these dyslexias: Friedmann and Rahamim (2007) and Keidar and Friedmann (2011) reported on individuals with LPD without attentional dyslexia and Friedmann et al. (2010b) reported on individuals with attentional dyslexia without LPD. Thus, this is an additional evidence that these dyslexias cannot stem from the same attentional source^5^.

When we think of *neglect dyslexia*, the imaginable connections to attention are different. One can think of neglect dyslexia as resulting from a deficit in orienting of attention to the left visual field. In fact, data from adults with acquired dyslexia already show that neglect dyslexia at the word level can appear without visuo-spatial neglect (Kinsbourne and Warrington, 1962; De Lacy Costello and Warrington, 1987; Patterson and Wilson, 1990; Haywood and Coltheart, 2001; see a summary and discussion in Cubelli et al., 1991 and Young et al., 1991). Such dissociation was also reported from Hebrew-speaking children and adolescents with developmental neglect dyslexia (Friedmann and Nachman-Katz, 2004; Nachman-Katz and Friedmann, 2007, 2010). The other direction of dissociation has also been reported: Primativo et al. (2013) reported seven patients with unilateral spatial neglect who did not have word-level neglect dyslexia. Such double dissociations already suggest that there are two different mechanisms underlying visuo-spatial neglect (and omissions of words on one side of text) and neglect dyslexia at the word level. The current results support these conclusions (as well as Haywood and Coltheart's, 2001 perception of word-level neglect dyslexia as separate from visuospatial neglect) from additional angle: Part A reported two individuals with developmental neglect dyslexia with no attention deficits, and 8 individuals with a deficit in orienting of attention without any dyslexia, including no neglect dyslexia. The results of part B include three additional individuals with developmental neglect dyslexia, none of whom had a deficit in orienting of attention and eleven participants with deficient orienting of attention who suffer from different types of dyslexia, none of which is neglect dyslexia.

Finally, let us consider the non-specific effect that a deficit in sustained attention may have on reading. One hypothesis we raised in the Introduction was that a deficit in *sustained* attention would cause a chain of events following which children will have more limited exposure to reading. In this case, many words will not be represented in the orthographic lexicon, and they will be read via grapheme-to-phoneme conversion, leading to *surface dyslexia*-like errors. This mechanism as a basis for surface dyslexia is also not supported by our results: we saw in part A 12 individuals who had surface dyslexia but no attention deficits, and 27 individuals who had attention deficits (including 19 with sustained attention deficit) without surface dyslexia. Part B added to these results by showing 15 individuals with surface dyslexia who had an attention deficit but intact sustained attention, and 12 individuals with sustained attention deficit, but without surface dyslexia. We also found results that do not support *executive* attention as the basis for surface dyslexia: we suggested that executive attention may be responsible for keeping the reader on the lexical route and resolving conflicts in the output buffer between inputs from the lexical and sublexical routes. However, this mechanism is not borne out, as in part A there were 12 individuals who had surface dyslexia but no attention deficits, and 27 individuals who had attention deficits (including 11 with executive attention deficit) without surface dyslexia. Part B added to these results by showing 20 participants with surface dyslexia who had an attention deficit but intact executive attention, and 10 individuals with executive attention deficit and dyslexia, but without surface dyslexia.

The other hypothesis, according to which sustained attention deficit would lead to a garden variety of errors in reading is also not supported by our results, especially given the 19 participants in part A who had sustained attention deficit but no dyslexia at all.

In addition, it was found that the different attention deficits are dissociable from one another (i.e., there were participants who were impaired in a single attention function). Most importantly, it seems that selective attention and orienting of attention—two spatial attention functions that sometimes are treated as interchangeable functions, are separate. We reported (in Tables 2, 3) 22 participants who showed no significant orienting deficit yet demonstrated selective attention deficit and 15 participants who showed the opposite pattern.

Thus, we saw double dissociations between letter position dyslexia and attention, including a double dissociation with selective attention; between attentional dyslexia and attention, including a double dissociation with selective attention; between word-based left neglect dyslexia and attention, including a double dissociation with orienting of attention; and between surface dyslexia and attention, including a double dissociation with sustained attention and with executive attention, as well as dissociations between vowel dyslexia and phonological dyslexia and attention. These results show that each of these dyslexias can occur with intact attention, indicating that attention deficits do not underlie these dyslexias. These results may suggest that the impairment in dyslexias such as letter position dyslexia, attentional dyslexia, or neglect dyslexia lies in an attention component that is specific to reading, an *orthographic-attention*. Such approach is also consistent with previous findings that describe letter migrations in words without digit migrations in numbers (Friedmann et al., 2010a); neglect of the left side of words without neglect of the left side of numbers (Friedmann and Nachman-Katz, 2004; Nachman-Katz and Friedmann, 2008); findings according to which MPH improves visuo-attention functions but not reading errors in letter position dyslexia or attentional dyslexia (Keidar and Friedmann, 2011); and findings according to which adults with developmental dyslexia perform poorer than controls on partial report task only in letter strings but not in symbol strings (Collis et al., 2013).

The differences between the findings of the current study and previous studies that reported comorbidities between reading and attention can be ascribed to several factors. Firstly, whereas previous studies focused on the group level and looked for correlations, we examined the question at the individual level and focused on the search for dissociations as a tool to examine whether attention deficit underlies dyslexia. Another difference relates to the level at which reading disorders were examined. We examined dyslexia and hence tested errors in reading at the single word (and nonword) level, whereas some of the previous studies that found relation between attention and reading tested reading speed, which may be affected by attention, and reading comprehension at the text level.

This research is the first to assess the intricate relations between types of dyslexia and types of attention deficits, and it has demonstrated how important it is to assess reading and attentional personal profiles of children and adults with reading and/or attention deficits. We have demonstrated that the different types of dyslexia are dissociated from the different attention deficits and that individuals who suffer from a reading disorder, attention deficit or both can be characterized by various reading and attention profiles. The sensitive identification of detailed reading and attention profiles may improve significantly the ability to select personalized tailor-made interventions that will aim at facilitating reading as well as other everyday functioning.

### Conflict of interest statement

The authors declare that the research was conducted in the absence of any commercial or financial relationships that could be construed as a potential conflict of interest.

## Figures and Tables

**Table A1:**
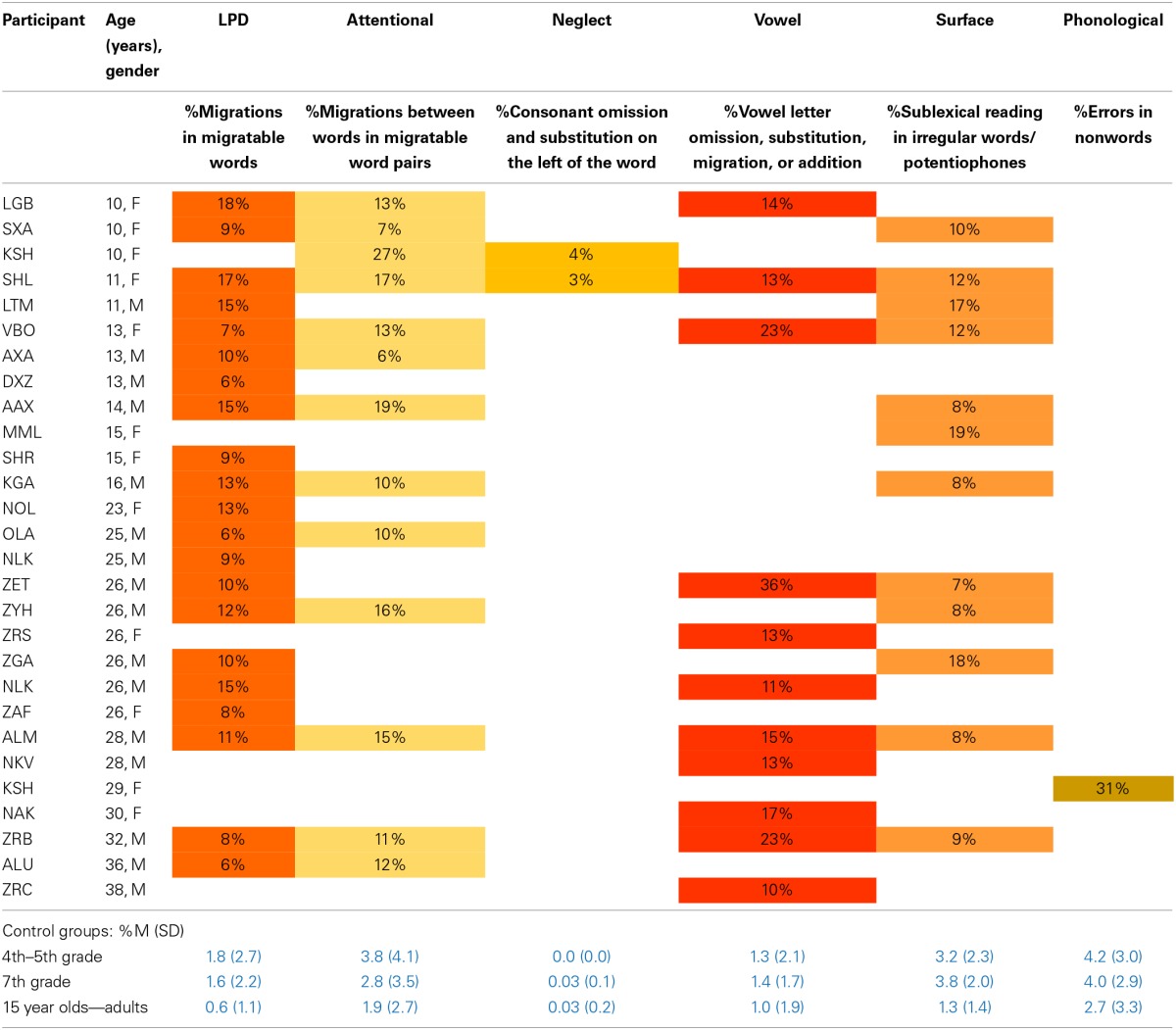
**Percentage of relevant errors in reading aloud of each of the 28 participants with dyslexia and with normal attention functions (summarized in Table 1)**.

*^a^All the error rates of the various types presented in the table are significantly larger than the age-matched control groups for this error type (p < 0.05), and indicate a dyslexia of the relevant type. Empty cells indicate that there were no relevant errors at all or that the relevant error rate was within the normal range, in most cases less than 1%. The percentages of errors that appear in the table for each dyslexia are: for LPD—migrations in migratable words, for attentional dyslexia—migrations between words in migratable word pairs, for neglect—consonant omission and substitution on the left of the word, for vowel dyslexia—vowel letter omission, substitution, migration, or addition for surface dyslexia—sublexical reading in irregular words and potentiophones, and for phonological buffer dyslexia—errors in non-words*.

*^b^The percentage of vowel errors of LGB, SHL, NLK, ALM, and NKV refer to their percentage errors in reading words, for the other vowel dyslexic participants the percentages are from nonwords*.

**Table B1:**
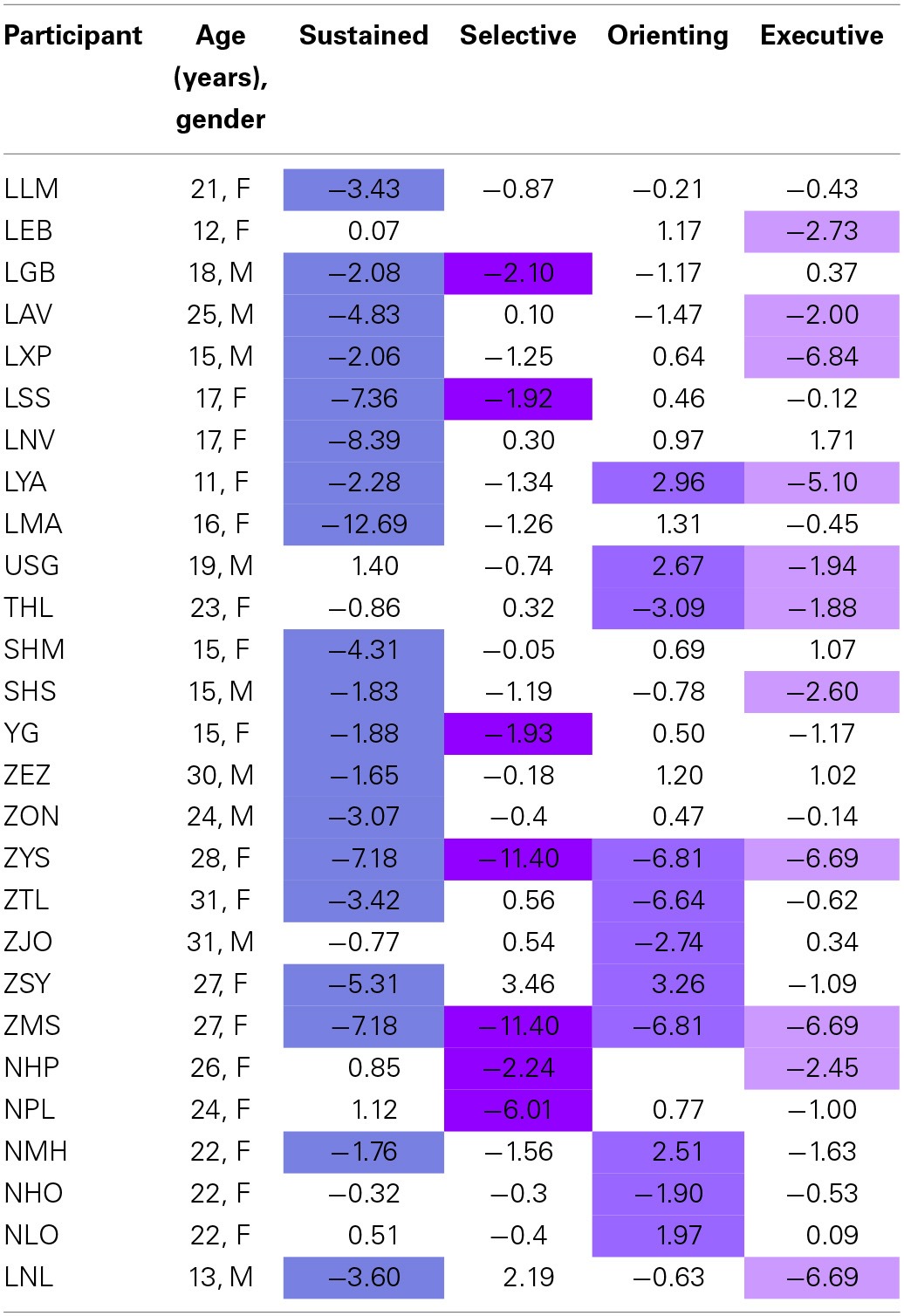
**Percentage of relevant z-scores in the attention tasks of each of the 27 participants with attention disorders and with normal reading (summarized in Table 2)**.

*LEB did not participate in a selective attention task; NHP did not complete the orienting attention task*.

## Footnotes

^2^In this study we focus on dyslexia, and hence on reading at the single word (and nonword) level. Another type of relation between reading and attention pertains to text reading and reading comprehension. Individuals with attention disorders may read an entire chapter and have no idea about its content (Levine, 1987), as a result they often have to read the same paragraph repeatedly in order to grasp its meaning (Robin, 1998, p. 284. See also Cherkes-Julkowski et al., 1995; Brock and Knapp, 1996; Stern and Shalev, 2013, for impairments of children with ADHD on comprehension at the text level).

^3^It is also possible that a deficit in sustained attention would induce a more general reading impairment, leading to garden variety of errors: letter substitution, addition, omission and migration, as well as possibly reading via grapheme-to-phoneme conversion.

^4^Notice that even if reduced attention span can account for surface dyslexia, it cannot account for other dyslexias, such as LPD, attentional dyslexia, and the others.

^5^The double dissociation between LPD and attentional dyslexia indicates that it cannot be the case that selective attention underlies both dyslexias but only one of them is affected in some cases because it is more sensitive to the attention deficit.

## Appendix C

**Table C1:** **Examples for the various types of Hebrew stimuli used in the reading tasks**.

**Stimulus type, the dyslexia it was used to detect, and the error type**	**Target word**	**Relevant error**
Migratable words—for letter position dyslexia (an example of a letter migration error)		
	mdnim	mdnim
	mad'anim	maadanim
	scientists	delicacies
Migratable nonwords—for letter position dyslexia and phonological dyslexia (an example of a letter migration error)		
	mrdgot	mdrgot
	mardegot	madregot
	nonword	stairs
Migratable word pair—for attentional dyslexia		
	xcb mgb	mcb xgb
	xacav magav	macav xagav
	squill wiper	situation grasshopper
Potentiophone—for surface dyslexia (an example of a sublexical reading)		
	Ktf	kTf
	katef	kataf
	shoulder	picked
Irregular word—for surface dyslexia (an example of a sublexical reading)		
	zat	zat
	zot	zat
	this	nonword
Words with a lexical potential for errors on the left—for neglect dyslexia (examples of letter errors on the left: substitution, addition, omission)		
	Slx	SlT/ Slxt/ Sl
	shalax	shelet/ shalaxt/ shel
	sent	sign/ you-sent/ of
Words with a lexical potential for vowel errors—for vowel dyslexia (examples of vowel letter migration, omission)		
	xlok	xolk/xlk
	xaluk	xolek/xalak
	gown	shares/smooth
Nonwords with a lexical potential for vowel errors—for vowel dyslexia (examples of vowel letter errors omission, substitution)		
	loSon	lSon/liSon
	lushon	lashon/ lishon
	nonword	tongue / to-sleep

*Each example shows the Hebrew target word and the word or words created by the relevant error, followed by orthographic transliteration, phonological transcription, and translation*.
